# The Surgical Diseases of Children

**Published:** 1861-05

**Authors:** 


ART. V.—*The Surgical Diseases of Children.* By J. COOPER FORSTER, Fellow of the Royal College of Surgeons, Surgeon to the Royal Infirmary for Children, etc. London: John W. Parker & Son, 1860. 8vo., pp. 348.

So marked a difference, in many points both of anatomy and physiology, exists between childhood and adult age, that, although the same laws are in force throughout both periods, their operation is necessarily modified with the physical changes undergone by the individual. In the child, the nervous system is highly impressible, the nutritive processes are actively carried on, the skeleton is in a transitional state, the sexual organs are undeveloped. After the second of the great changes which affect both sexes alike, adolescence establishes a much more even balance between the several constituent systems of the body; and the progress of their maturation is from that time more steady, uniform, and equable.

Previous to puberty, therefore, not only are some affections met with from which adults are more or less completely exempt, but those injuries and diseases which are common to all periods of life are modified, in their symptoms, course, diagnosis, prognosis, and in the treatment demanded, by the peculiarities of childhood. The existence of such works as those of Barthez and Rilliet, Evanson and Maunsell, Condie, Meigs, West, Underwood, and others, shows the extent and importance of the medical branch of practice among children; and we think that almost every one must have felt from time to time the want of a corresponding literature in the department of surgery. It is true, that in most of the systematic treatises on general surgery much of the matter appropriate to such a book as that before us may be found, but it is there introduced incidentally. An examination of several such works, of deservedly high rank, has impressed us with surprise at the degree to which these very important topics are neglected; the operations practiced on children are described and explained, but the antecedent and more scientific subjects we have alluded to are passed over almost in silence.

So far as we are aware, this volume of Mr. Forster's is the first essay at filling up the before-mentioned gap in surgical literature. His extensive experience at the Royal Infirmary for Sick Children, as well as that world-renowned institution, Guy's Hospital, must give his views a large share of authority; more than three hundred children, we are told, are annually admitted into the surgical wards of the latter charity alone. In his preface, Mr. Forster states that his work is based entirely upon his own observations, and that it is wholly original; he also says that brevity has been a constant object with him. Let us remark here, that in the excess of these virtues we find the only faults of his book. Had he extended his limits so as to embrace the literature of his subject, the various theories, plans of treatment, etc., defended by other writers, and confined himself less rigorously to the task of producing as short a book as possible, we think he would have done better both by himself and by his readers. At the same time, we think that Mr. Forster has done a most important work, not only in opening a new field, but in publishing the results of his labors; and we are disposed to receive his contribution most thankfully.

No branch of surgery has been more benefited by the introduction of anæsthetics, than that now in question. A crying, struggling child presents a most difficult subject either for examination or operation, to say nothing of the after effects, physical and moral, of the contest. Chloroform is safely employed in children, unless some organic disease of the brain, heart, or lungs is present. Mr. Forster thinks local anæsthesia, by means of freezing mixtures, more useful in the later years of childhood than in infancy; and even then it acts but superficially.

In speaking of hare-lip, Mr. Forster gives a very clear description of the operation, which he thinks should be performed as early as possible, in order to facilitate sucking. He very properly prefers the knife to the scissors; but we have found it more convenient to employ a small spade-shaped wooden spatula to cut down upon, than to trust to forceps for confining the edge of the fissure. A pair of wooden forceps with one broad blade might be used with advantage. Mr. Forster also advocates the continued suture for keeping the pared edges together, alleging that it leaves less of a mark than pins do; we have always been disposed to prefer the quilled suture to any other means of coaptation, from the fact of the general support it gives to the tissues. A very fine wire may be used to make the points with, and the marks thus left can hardly be more observable than those from the continued suture.

Passing on to Chapter V., on affections of the pharynx and œsophagus, we find a most interesting case related, in which gastrotomy was resorted to for the relief of contraction of the œsophagus from corrosive poison. A boy four years and four months of age came under Dr. Addison's care at Guy's Hospital, having swallowed, seventeen weeks previously, what was supposed to have been a solution of potash. Fifteen weeks after the accident, after having been free from symptoms for more than three months, he began again to suffer from dysphagia, and at the time of his admission was quite low from want of nourishment. Nine days later, the case being desperate, an incision two inches long was made in the left hypochondriac region, an opening effected in the stomach, and the edges secured by the continued suture to part of the parietal wound; the rest of the latter orifice was closed by the same means. A large vessel in the wall of the stomach was divided, and both ends of it had to be tied. Chloroform was used to produce anæsthesia. Food was immediately given by the gastric fistula, and the child got along very well for nearly three days, when he suddenly complained of great abdominal pain, became collapsed, and died in four hours. At the autopsy, the peritonitis (to which death was evidently due) was found to have arisen from the giving way of some of the sutures, and the consequent escape of food into the peritoneal cavity. An excellent wood-cut shows the post-mortem appearance of the body.

Mr. Forster passes over intestinal obstructions in silence; but we think that the above case would have justified him in speaking of them, and in recommending what we should certainly propose where any clue was afforded to the seat of the difficulty—an operation analogous to the above. If death is inevitable, which in such cases may often be asserted, the results of observation of wounds of the abdomen would warrant us in proceeding, at once, boldly, but cautiously, to attempt relief by gastrotomy, as the sole though desperate resource of art.

In Chapter VII., on diseases of the trunk, we notice some interesting remarks on vascular growths from the umbilicus, as well as on fæcal fistules at this point. The treatment in the former affection consists simply in ligation; in the latter, in paring the edges of the opening, and bringing them together by a hare-lip or quilled suture.

Vesical calculus, so common in boys, is of very rare occurrence in female children. In the former, Mr. Forster prefers lithotomy, by the ordinary lateral method; dwelling particularly on the importance, on account of the smallness of the prostate at this period of life, of tearing rather than cutting that organ. Hemorrhage of any moment he has never met with in this operation, except in boys near puberty, and in such cases he would defer surgical interference until the organs were fully developed. The best treatment in the case of girls is by lithotrity, unless the stone be very hard; and then a combination of dilatation of the urethra with incision of the neck of the bladder may be resorted to.

The subject of nævus occupies two chapters of Mr. Forster's book; and is illustrated by a number of very excellent colored lithographs. Formations of this kind are classified as cutaneous, subcutaneous, and mixed; they are defined as growths similar in structure to erectile tumors, but "not endowed with the power of filling themselves with blood." To this latter statement we take exception; no part has the power of filling itself with blood, unless it is in some way susceptible of special stimulation, like the nipple or the penis. Nævi do not, so far as we know, contain nerve-fibres, or any active tissue-element; they become the seat of passive congestion only, when the adjacent parts are distended with blood, as when a child cries violently. Mr. Forster looks upon the ligature as the best and surest mode of treatment for nævus, except when the disease is wholly subcutaneous, in which case he always resorts to the injection of a solution of the perchloride of iron. He remarks that many small nævi may be tied very tightly, the pins used for transfixion removed at once, and the ligature in a few hours afterward, with perfect success; in larger growths of the kind, however, it is necessary to maintain the constriction until sloughing has begun. The various other plans of treatment which have been proposed are fully discussed—excision, pressure, ligation of vessels of supply, vaccination, irritant and escharotic applications, introduction of caustic, seton, actual cautery, needles and twisted sutures; of all these, not one is adapted to any large number of cases. Vaccination may be very advantageously used in small nævi on very young children, but we have never seen it succeed under any other conditions.

In view of the very great importance and interest of the subject of injuries and diseases of the bones and joints in children, we think Mr. Forster has left a vast deal unsaid which his experience must have taught him in regard to these affections. As to fractures and dislocations occurring in children, they differ from those in adults mainly in their therapeutics; and every surgeon who has much to do with children acquires "wrinkles," as the boys say, which would be of service if communicated to the profession. The slight purchase which short and small limbs afford for a confining apparatus, the extreme restlessness so common in early life, and the eel-like facility with which our little patients so often contrive to worm themselves out of what seems at first to be an efficient dressing, make it extremely desirable that the best plans of treatment devised should be generally known.

Upon the diseases of these organs, our author has spoken somewhat more fully. He advocates entire rest in coxalgia, of which he gives several interesting cases, and speaks also of the value of tonics; but he does not seem to us to say enough of gentle purgation, which we think an important element of the treatment. In this connection we cannot help alluding to a case under our charge four or five years ago, in which a girl about seven years old had had all the symptoms of the disease, gradually increasing in severity for more than a year. All attempts at confining her limb were futile, and we had to be content with the steady employment of tonic and laxative medicines; under which she recovered so entirely as to retain only a slight limp in walking or running.

Resection of the head of the femur does not seem to Mr. Forster to satisfy the expectations which its theoretical consideration would excite; and such would seem to be the legitimate conclusion from the few experiments with it which have as yet been made in this country.

In speaking of foreign bodies in the ear, Mr. Forster says:—

> "The more the meatus is pulled about by instruments, the less likely is the removal of the foreign body to be effected. Syringing with warm water is therefore the practice to be adopted. When the foreign body has been thus destroyed, the end of a curette may be sometimes useful to remove it from the orifice; but even then any attempts which give pain to the patient should be at once discontinued. Repeated syringing may be required, as no force should be used, even if unsuccessful in the first or second attempts at extraction. If the foreign body cannot be removed by syringing, which, unless there has been previous injudicious interference, is seldom the case, it should be left to ulcerate its way out."

Although deprecating the use of any unnecessary force, we are sure that the best practice in such a case would be to persevere in the use of instruments, drilling or breaking up the foreign body. About two years since we were called to see a boy six or seven years old, who had had a piece of slate-pencil, five-eighths of an inch long, forced into the meatus by a schoolmate; and so firmly imbedded was it across the upper end of the channel, as to be entirely immovable. Feeling sure that if allowed to remain it would cause inflammation, and perhaps the total loss of hearing, we administered ether, and then, with a strong probe curved at the end, pried the piece of pencil into the axis of the meatus, so that it could be removed with forceps. The change of position was not effected without the exercise of very great force, but no unpleasant symptom followed, and in a few days the boy was quite well. In such a case as this it would be manifestly absurd to rely upon syringing the meatus, and equally improper to leave a hard mass resting in close proximity to the membrana tympani; so that, had any greater force been necessary for its dislodgement, we would have proceeded to attempt breaking it up, by drilling it with a dentist's file.

When speaking of spina bifida, Mr. Forster discourages surgical interference, whether by incision, ligature, or injection; recommending simply mechanical support of the tumor. The other malformations usually met with in children at birth, club-foot, imperforate anus, etc., are quite summarily dismissed.

A few words on tetanus, and a section on burns and scalds, conclude this volume. The former affection, the details of several cases of which are given, Mr. Forster thinks is not always consequent upon, but may be merely coincident with the wounds which are supposed to give rise to it. He would treat it by means of anæsthesia, anodynes, support, and wine. For burns and scalds he recommends the use in the first place of raw cotton, and subsequently of ointments, such as that of the oxide of zinc, or resin cerate. Raw cotton is undoubtedly a very comforting application in these cases, where the cuticle has not been destroyed; but for seven or eight years past we have preferred fresh lard, thickened if necessary with simple cerate, to every other form of dressing for burns. We speak advisedly when we say, that we have substituted this for other remedies in hundreds of cases, and never yet knew it to fail in quieting pain and promoting the healing process. Its advantages we believe to consist in its unirritating nature, and in the complete protection it affords from the air.

We have now passed in review the most important of the doctrines inculcated in Mr. Forster's book. Its meagerness, as we have said, is its only fault in our eyes; and this we attribute partly to the author's modesty and partly to his desire to write strictly from his own observation. We should be glad to find his next edition, which, we trust, may soon be called for, augmented to three or four times the size of this, and furnished with his views, in full, on many subjects which he has now passed over hastily. A man with his practical tendencies, clear head, and abundant experience, may write at length without fearing the charge of diffuseness; and we would remind him that no man can take up any special department in medicine or surgery without going over ground already in a manner covered by general treatises.

Unusual liberality has been displayed by Messrs. Parker & Son in the getting up of this very handsome volume. Both its illustrations and its letter-press are exquisite, and its typography is altogether correct.

